# A Spreading Concern: Inhalational Health Effects of Mold

**DOI:** 10.1289/ehp.115-a300

**Published:** 2007-06

**Authors:** Bob Weinhold

The issue of mold contamination has drawn the national and international spotlight on the heels of publicity about prominent situations, such as a hotly contended link between mold and severe illness—and one death—in 10 Ohio infants in 1993 and 1994; a major 2001 insurance battle over the moldy Dripping Springs, Texas, house of Melinda Ballard and her family; the mushrooming mold infestations indoors and out along the Gulf Coast after Hurricanes Katrina and Rita slammed ashore in 2005; and the mold infestation that helped spur the February 2007 outcry over the treatment given to recuperating soldiers at Walter Reed Army Medical Center. As recently as 25 years ago, inhaled mold was considered primarily a nuisance, not a serious health threat. But the growing scientific and medical evidence suggests the threat is widespread and, for some people, quite serious.

In the 9 June 2006 report *Mold Prevention Strategies and Possible Health Effects in the Aftermath of Hurricanes and Major Floods*, the CDC concluded that “excessive exposure to mold-contaminated materials can cause adverse health effects in susceptible persons regardless of the type of mold or the extent of contamination.” The CDC based some of its findings on a landmark 2004 report, *Damp Indoor Spaces and Health,* by the Institute of Medicine (IOM) of the National Academies. Relying on the IOM report, and dozens of studies and reports that have been published since, many organizations and individuals that must deal regularly with mold problems have begun to take steps to reduce the threat.

But many of the puzzle pieces—exactly who is vulnerable, to what extent, and under what conditions—are still missing. The vast information gaps that remain continue to feed significant controversy in the legal, insurance, political, scientific, medical, public health, and building design, construction, management, and maintenance arenas.

## Growing Suspicion

Of the 100,000 or so known fungal species found on the planet, about 500 species are currently thought to be harmful to people, according to the CDC. Some of those that pose ingestion threats (such as *Aspergillus*, via contaminated grains and nuts) or skin infection threats (such as *Trichophyton*, which causes athlete’s foot) have been either well-recognized or strongly suspected for years, even centuries. As for inhalational threats, although molds such as *Stachybotrys* and *Aspergillus* have received perhaps the most popular attention, scientists are not yet sure which species may be the worst for human health.

A few threats from inhaled molds have been perceived for a long time. Since the 1890s, outdoor settings in the U.S. Southwest have been linked with coccidioidomycosis, caused by a fungus in the soil. At least 50 years ago, there were some indications of mold-related health problems in agricultural and certain occupational settings, causing illnesses such as pneumomycotoxicosis. In areas near rivers in the central United States, a fungus has been known for at least 30 years to cause blastomycosis. By about 25 years ago, there was some initial evidence that damp indoor spaces were linked with health problems such as bronchitis, asthma, cough, wheeze, and shortness of breath. But there has been little specific knowledge until recently.

Wherever they grow, molds must have some source of water and food. The accumulating evidence has shown that problems with mold can surface anywhere in the world after just one or two days of moisture exposure, in settings wet or dry, hot or cold, north or south. The same conditions that give rise to mold growth also support many bacteria. Many components and emissions from these fungi and bacteria are known or suspected to harm human health. Mycotoxins, which are secondary fungal metabolites, have been one primary focus, and more than 180 have already been identified. Other components of fungi or bacteria in damp spaces that are known or suspected to pose a threat include volatile organic compounds, live or dead spores, fragments such as beta glucans, and numerous allergens.

Mycotoxins have often been the main point of contention in recent insurance claims and lawsuits over suspected harm from moldy buildings. In the 2004 EPA-funded report *Guidance for Clinicians on the Recognition and Management of Health Effects Related to Mold Exposure and Moisture Indoors*, researchers at the Center for Indoor Environments and Health at the University of Connecticut Health Center wrote that mycotoxins can elicit responses in almost anyone they come in contact with, that the health effects are worrisome, and that infants, at least, should be removed from suspect settings.

After reviewing the evidence available by 2004, the IOM concluded there are moderately strong or at least limited links between damp indoor spaces and a handful of health problems, such as asthma, cough, wheeze, hypersensitivity pneumonitis, and a range of other upper and lower respiratory problems. For other health problems under suspicion based on many anecdotal accounts and limited scientific and medical evidence, such as headache, memory loss, nausea, diarrhea, diabetes, fatigue, and fever, the lack of incontrovertible evidence was typically due to a lack of rigorous research, not because of studies that conclusively disproved a connection.

Among the weaknesses the IOM notes in many current studies is a tendency to use self-reported visual or odor presence of mold, instead of actual measurments of some kind, and little consideration of multiple exposures, including additive or synergistic effects. In addition, the committee noted that its findings did not address people with compromised immune systems.

## Why Now?

It is likely that building dampness and mold have caused widespread but largely unrecognized adverse respiratory health effects for centuries, says William Fisk, acting division director for the Environmental Energy Technology Division at the Lawrence Berkeley National Laboratory. But the increasing immune-compromised population around the world may be one reason why health problems from inhaled mold and bacteria appear to be on the rise recently. Population growth, higher percentage of elderly, emerging diseases such as HIV, and increases in smoking and in many chronic illnesses (often for unknown reasons) are only a few of the reasons that, compared to just a century ago, there are hundreds of millions more people with weak or stressed immune systems. The CDC has identified many immunocompromised subpopulations, as well as pregnant women, as being potentially more vulnerable to exposures in damp indoor spaces.

In addition, the dramatic increase in the percentage of people living in urban areas may be playing a part. Researchers at Hospital General Universitario Gregorio Maranon in Spain reported in the June 2006 issue of *Medical Mycology* that *Aspergillus* spores in outdoor air are more common in urban than rural settings in the province of Madrid. Worldwide population increases have also pushed more people into wetter settings, such as coastal and riparian floodplains, other bottomlands, and hurricane-prone areas.

Other risk factors arise from modern building practices, conveniences, and shortcuts. Poorly built flat roofs cannot shed rain-water, while venting clothes dryers indoors can direct moist air to vulnerable interior surfaces. Tighter building envelopes in modern homes slow the escape of water vapor associated with bathing, cooking, and even breathing; newer homes also have insulation-filled cavities that dry slowly after the inevitable small leaks. Further, the tight seal on newer housing may exacerbate problems during the heating of buildings, when humid indoor air contacts cold walls or windows (although the reverse is true for an air-conditioned building when it is hot outdoors). Also, there are many anecdotal reports that molds grow more readily on the paper-coated surfaces of modern wallboard than on older plaster walls. A few companies have introduced wallboard products they say are more resistant to mold growth, but some critics say these products still may support mold in settings that routinely get wet, such as kitchens, bathrooms, or areas with leaks of some type.

The substantial increase in air conditioning all over the world is another potential culprit, with more than fifteen studies consistently indicating a strong link with numerous respiratory symptoms, says Fisk. Microbes thriving in air conditioning systems, including fungi and bacteria, likely contribute to that link, he says.

Buildings have often been constructed without sufficient attention paid to indoor water problems. In an assessment of health and economic impacts of dampness and mold published in the June 2007 issue of *Indoor Air*, Fisk and EPA indoor environment specialist David Mudarri found that approximately 47% of U.S. homes have dampness or mold problems. Their review of other studies led them to conclude that schools, offices, and institutional buildings have similar problems. The EPA Building Assessment Survey and Evaluation Study of 100 randomly selected U.S. office buildings supports that conclusion, with its finding, reported at the 2002 9th International Conference on Indoor Air Quality and Climate, that 45% had ongoing water damage problems. University of Cincinnati environmental health professor Tiina Reponen and her colleagues noted in a May 2006 study in the *Journal of Occupational and Environmental Hygiene* that the percentage of buildings of all types that have mold contamination is likely much higher in tropical and subtropical settings.

## Cracking the Mold Code

Many new studies have provided additional evidence that mold likely deserves serious attention. Fisk and Mudarri demonstrated in their June 2007 assessment that 21% of current U.S. asthma cases may be attributable to dampness and mold in homes, with schools, offices, and institutional buildings playing a similar unhealthy role. In a companion meta-analysis of 33 studies also published in the June 2007 issue of *Indoor Air*, Fisk and Berkeley Laboratory colleagues found that dampness and mold exposures increase the occurrence of a range of respiratory problems by 30–50%.

Many other examples of potentially significant findings have been published in the past three years. In the May 2004 issue of *EHP*, Kati Huttunen of the Finnish National Public Health Institute and colleagues demonstrated synergism between various indoor fungi and the bacterium *Streptomyces californicus*, including increases in production of tumor necrosis factor–α and interleukin-6 in various circumstances. In the February 2006 issue of *Toxicology and Applied Pharmacology*, a Michigan State University team described exacerbated damage when exposure to a mycotoxin was preceded by exposure to a bacterial fragment, in this case the endotoxin component lipopolysaccharide. More detailed knowledge of the wide-ranging olfactory system damage that a mycotoxin can wreak appeared in the July 2006 issue of *EHP*, and in the following month’s issue, Case Western Reserve University researchers described how they identified potential biomarkers of mycotoxin exposure. An article in the 3 June 2007 issue of *Toxicology* addresses elucidation by a second Finnish team of specific accelerated genotoxic and cytotoxic damage by a cultivated fungus–bacterium mixture.

EPA research biologist Stephen Vesper and colleagues have performed a series of experiments to develop better methodology for predicting mold exposure risk. After almost a decade and a half of work, they have created a Relative Moldiness Index that uses quantitative polymerase chain reaction to measure concentrations of 36 indicator mold species present in floor dust samples taken inside a building. This standardized analysis, described in the January 2007 issue of the *Journal of Exposure Science and Environmental Epidemiology*, is used to indicate the amount of water damage in a home, providing more accurate exposure information that may help to predict health problems. They expect to soon publish information about its successor, the Environmental Relative Moldiness Index, which covers more buildings in more geographic settings, and benefits from improved sampling protocols and analysis of information.

As researchers explore the potential contributions of damp conditions to human health problems, they’ll need to be careful about exactly which test animals they use. Several reports, such as a Harvard study in the October 2006 *American Journal of Respiratory Cell and Molecular Biology*, have shown that different mouse strains vary significantly in their biological responses to a tested fungus. In addition, scientists face the usual uncertainties inherent in extrapolating results from any animal testing to humans.

## Not Messing Around

Until very recently, building design was not widely acknowledged as an important factor in preventing water problems. As recently as 2005, the American Institute of Architects (AIA) emphasized in an issue brief to its members that mold problems are tied to maintenance of a building’s plumbing and ventilation systems, not the initial building design. Just a year later, however, an article in the 29 September 2006 edition of the AIA publication *AIArchitect* emphasized that design details are critical in preventing mold problems. Some of the points of vulnerability highlighted included roof underlayments, concrete foundation sealants, flashing around windows and doors, and grading around the building.

Many contractors also are paying more attention. “We’ve told builders to be vigilant about moisture issues in all stages and to treat it seriously,” says David Jaffe, vice president of construction liability and legal research with the National Association of Home Builders. But problems still occur, he acknowledges, citing the continuing stream of insurance claims and lawsuits over mold concerns in both residential and nonresidential buildings: “It’s an ongoing issue. We’re always looking for ways to improve.” Other organizations, such as the American College of Occupational and Environmental Medicine and the American College of Medical Toxicology, remain skeptical that mold poses a serious threat to more than a small number of people.

Doubts about mold threats, uncertainty over who should be responsible for problems that may arise, and variable guidance on appropriate remediation continue to play a role in political responses to mold concerns. At least 46 states and the District of Columbia have approved some type of insurance coverage limitation for residential policies, and such exclusions are becoming more common for commercial properties, says Michael Barry, director of media relations for the U.S. Insurance Information Institute. According to the National Conference of State Legislatures, since 2001 at least 31 states have approved, have rejected, or continue to consider laws that address mold problems in some way, such as contractor liability, real estate agent or landlord liability for disclosure, or licensing of mold inspectors, testers, and remediators.

Given the evidence at hand, Health Canada has determined that mold may pose a health hazard, and on 31 March 2007 released brief recommendations for cleaning up mold in residences. The EPA is developing guidelines for moisture control “best practices” in all phases of design, construction, and maintenance, and may finalize the guidelines in 2008, says Laura Kolb, an environmental health scientist with the agency’s Indoor Environments Division.

Much more information continues to surface through research and public health efforts around the world, and there is some communication among various groups. But “not much has been done to move the science forward that’s applicable to broad populations,” says Allison Stock, a toxicologist with the CDC’s National Center for Environmental Health. One roadblock may be that there is no concerted, coordinated national or international effort to address the dozens of information gaps identified in the IOM report.

Given such shortcomings, “We’re still quite some way from being able to set [exposure and remediation] standards,” says Marsha Ward, a principal investigator in the EPA Immunotoxicology Branch. In the interim, groups such as the Restoration Industry Association are giving it their best shot and updating remediation guidelines for their members, targeting completion by 2008, says communications director Patricia Harman.

Another critical area requiring attention is the very limited repertoire of effective medical treatments to prevent illness or treat people experiencing certain ill effects such as allergic bronchopulmonary aspergillosis and acute idiopathic pulmonary hemorrhage, says Lynnette Mazur, a professor of pediatrics at the University of Texas Medical School at Houston and coauthor of a 6 December 2006 *Pediatrics* policy statement on noninfectious health effects from molds. Mazur points out that with respect to allergic rhinitis and asthma, however, there are very effective environmental and pharmacological treatments available.

Regardless of all the remaining uncertainties, the overall recommendations of many organizations and agencies worldwide are reaching a common conclusion: Don’t mess with mold. If you can see or smell it—and especially if health problems are occurring—clean it out, throw it out, or get out.

## Figures and Tables

**Figure:**
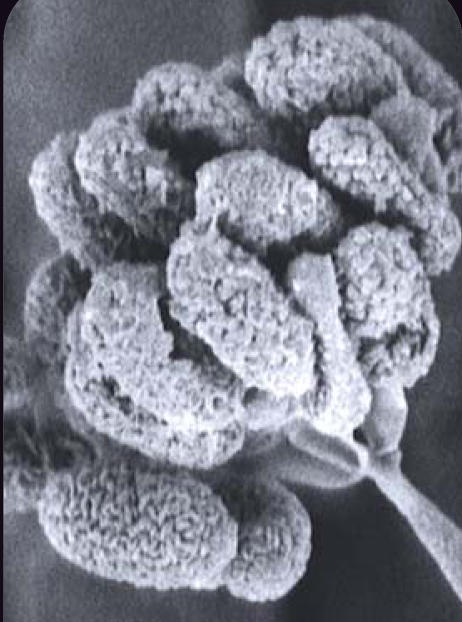
Mold on the move Recent high-profile news reports have raised awareness of the possible threats posed by indoor molds such as *Stachybotrys chartarum* (above), also known as *S. atra*.

**Figure:**
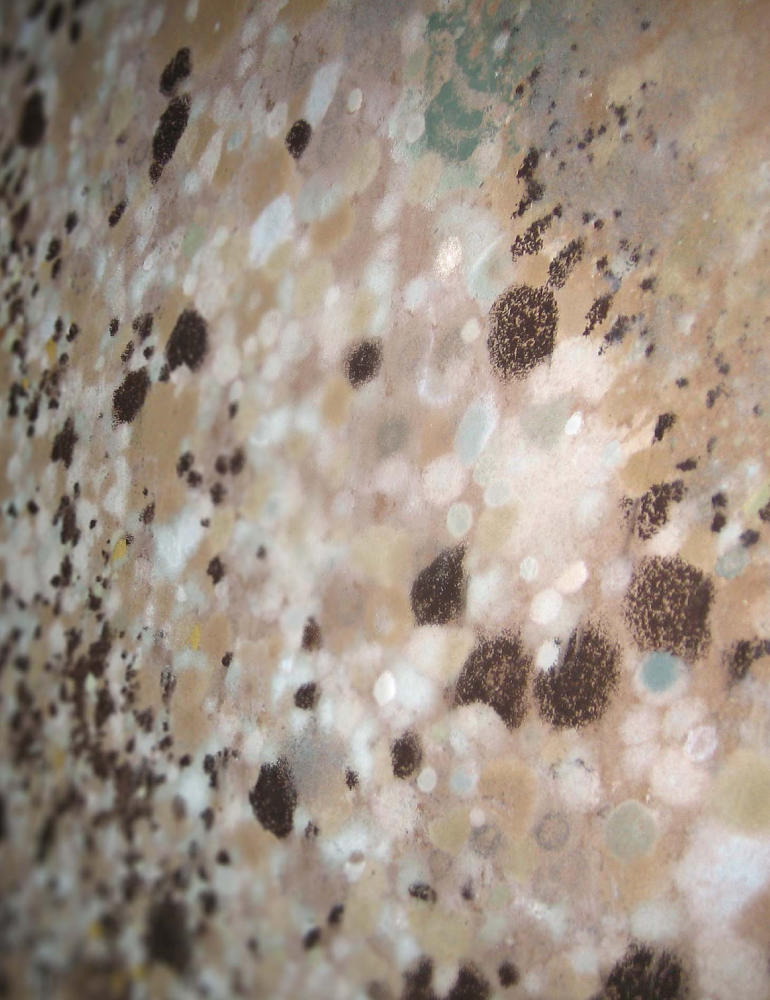


**Figure:**
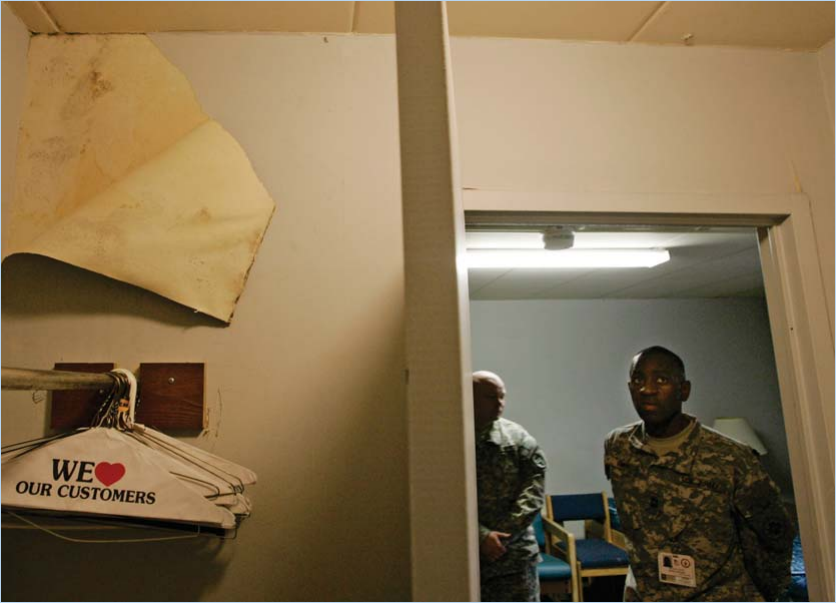
Bad scene A section of wallpaper is pulled back to reveal mold in Building 18 of the Walter Reed Army Medical Center. Indoor mold and the bacteria that often grow with it are believed to pose a greater threat to immunocompromised people.

**Figure:**
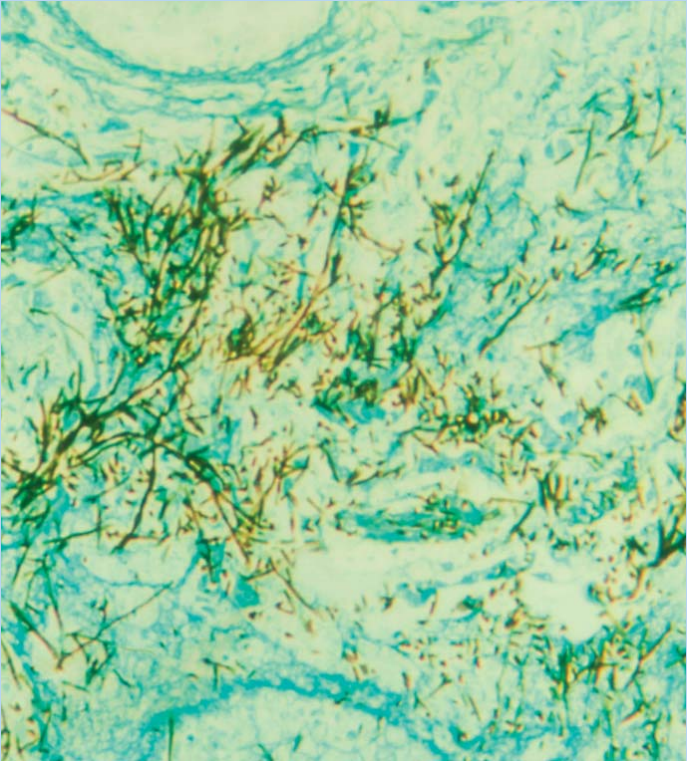
Inner workings Light micrograph shows a section of a human lung tisse (blue) embedded with *Aspergillus* (dark brown). Aspergillosis is caused by the inhalation of fungal spores, which are usually present in the air. In healthy people, the immune system destroys the spores before they cause any harm. In those with a weakened immune system, however, the infection is potentially fatal.

**Figure:**
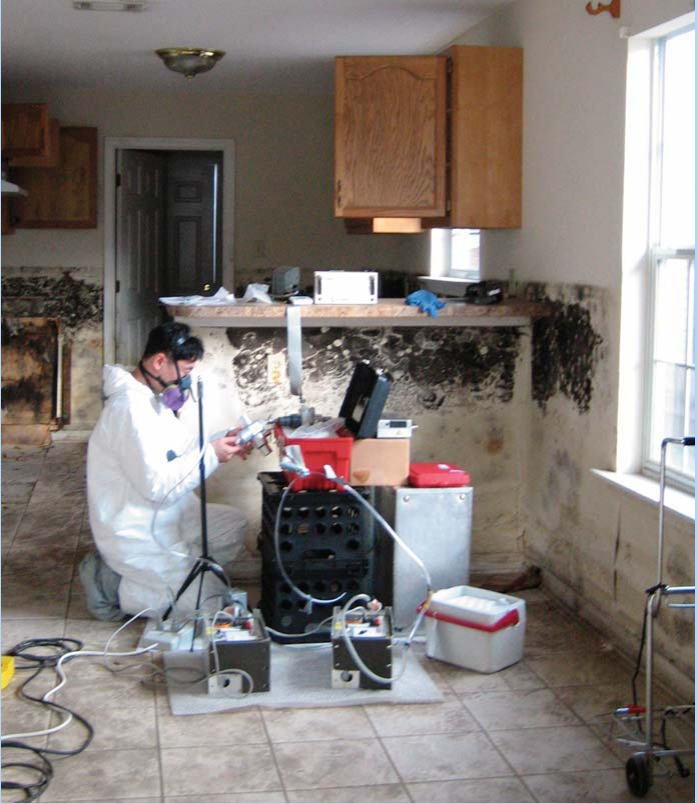
Home-based health threat A University of Cinncinnati researcher prepares to sample air inside a moldy Gulf Coast home that was flooded in the 2005 hurricane season.

